# Predictors of positive or negative reactions to learning Alzheimer’s biomarker results

**DOI:** 10.1093/geroni/igab046.3426

**Published:** 2021-12-17

**Authors:** Claire Erickson, Nate Chin, Erin Jonaitis, Fred Ketchum, Carey Gleason, Lindsay Clark

**Affiliations:** 1 University of Wisconsin-Madison, Madison, Wisconsin, United States; 2 UW Madison, Madison, Wisconsin, United States

## Abstract

With improved detection of Alzheimer’s disease and biomarker accessibility, more adults with no or mild symptoms may learn their AD biomarker results. Yet, potential psychosocial impact of learning AD biomarkers is not well understood. In a phone survey, we assessed potential reactions after learning about a hypothetical positive AD biomarker result. Data were collected from cognitively healthy participants (n=334, mean age=64.8±7.7) enrolled in longitudinal AD studies. Exploratory factor analysis identified five latent factors following a hypothetical positive biomarker result: advanced care planning, lifestyle changes to reduce dementia risk factors, psychological distress, subjective cognitive complaints, and stigma. Using linear regression, we found that predictors of potential pessimistic reactions (distress, cognitive complaints, stigma) included higher trust in research (Distress:b:0.04, p:0.04), no dementia family history (Stigma:b:-0.30,p:0.04), poorer memory self-rating (Cognitive complaints:b:-0.19,p:0.02), and Black racial identity (Cognitive complaints:b:0.30,p:0.02, Stigma:b:0.40,p:0.003). Predictors of potential optimistic reactions (advanced care planning, lifestyle changes) included more trust in research (Planning:b:0.07,p<0.0001) and Black racial identity (Planning:b:0.38,p:0.003), as well as younger age (Lifestyle:b:-0.02,p:0.02) and belief in AD controllability (Planning:b:0.22,p:0.003, Lifestyle:b:0.23,p:0.002). Concern about developing AD was associated with increased likelihood of all potential reactions. While AD concern associates with optimistic and pessimistic potential reactions, specific factors of family history, racial identity, trust, belief in AD controllability, and memory rating differentially predict each of the potential outcomes of learning AD biomarker results. These findings may help target education efforts to prepare and reduce risk of negative reactions for cognitively healthy adults who learn their AD biomarker results.

